# Is It Rational to Study Coagulations Test Routinely before Operations and Invasive Procedure: Single Center Retrospective Study

**Published:** 2019-07-01

**Authors:** Fergün Yılmaz, Tuğçe Karslı, Demet Kiper, Fusun Gediz, Bahriye Payzın

**Affiliations:** 1 Department of Hematology, Marmara University, Istanbul, Turkey; 2 Department of Hematology, Izmir Katip Celebi University, Izmir, Turkey; 3 Department of Hematology, Izmir Bozyaka Training and Research Hospital, Izmir, Turkey

**Keywords:** Coagulation test, Prothrombin time (PT), International normalized ratio (INR), Activated partial thromboplastin time (APTT), Preoperative

## Abstract

**Background:** Detailed history taking, physical examination and laboratory tests are useful tools to document any abnormal bleeding risk before an operation or an invasive procedure. Although coagulation tests are routinely used to demonstrate the pathological situations at the coagulation cascade or to follow-up the anticoagulation therapies, their role in determining the bleeding risk in preoperative patients is controversial.

**Materials and Methods**: In this study, we aimed to evaluate the patients referring to our hematology clinic at Izmir Katip Celebi University Hospital for preoperative consultation due to elevated levels of coagulation tests.

**Results:** Fifty-six patients with high PT/PTT levels were enrolled in this study. Twenty-six (46.4%) patients were male and 30 (53.6%) were female. The median age was 34 (18-75) years. We documented bleeding history in 12 (21.4%) patients. The patients having a bleeding history revealed mostly abnormal uterine bleeding, epistaxis, and gingival bleeding. Life threatening bleeding was not reported in any of the patients.  The operations were cancelled or postponed at least one month in 38 (67.8%) and 10 (17.8%) patients, respectively. Per-operative or post-operative abnormal bleeding was not documented. We did not find any statistically significant difference between groups with or without elevated coagulation tests in terms of abnormal bleeding in the operations.

**Conclusion:** Coagulations tests should be studied in selected group of patients. Additionally, mildly elevated results should be interpreted carefully to decrease the rate of cancellation and delay in operations and unnecessary increase in costs.

## Introduction

 One of the major complications during or after an operation is abnormal bleeding. It is important to find out any pre-existing coagulopathy to minimize the risk of bleeding because uncontrolled coagulopathy may result in life-threatening bleeding and increased blood transfusion. Detailed history taking, physical examination, and laboratory tests are useful tools to document any abnormal bleeding risk before an operation or an invasive procedure^1^^ -^^3^. Guidelines emphasize the importance of history taking, focusing on personal and family history of abnormal bleeding ^2^^, ^^3^. In a prospective multi- center study in 17 centers, 2498 patients were evaluated. In this study, it was reported that the fatal bleeding risk was 0.13 % in patients who did not reveal any bleeding risk during history and physical examination^4^. Although in the literature there are questionnaires to document the preoperative bleeding risk ^1^^, ^^5^^, ^^6^, there are very limited validated questionnaires, which is suitable for every day practice in Turkey. 

Under these circumstances and in the absence of well-constructed questionnaire, coagulation tests become the mainstay in clinical practice for defining the high-risk patients. 

In clinical practice, prothrombin time (PT), international normalization value (INR) and activated prothrombin time (a PTT) are most commonly used coagulation tests. Although these tests are routinely used to demonstrate the pathological situations at the coagulation cascade, to diagnose factor deficiencies and follow-up the anticoagulation therapies ^7^^, ^^8^, their role in determining the bleeding risk in preoperative patients is controversial ^9^^-^^11^. In a systematic review by Munro et al., abnormal values for PT and aPTT were reported in only 4.8 % and 15.6 % of the patients, respectively when these tests were routinely used before operations to determine the risk of bleeding. In this study, it was also reported that elevated PT or aPTT levels are rarely related to change in patient management^12^. Moreover, when these tests are routinely used in the preoperative setting, it is very difficult to interpret the mildly elevated levels in a patient who has no other risk factors for bleeding. These results may lead to delay in an elective surgery or an invasive procedure and also in an increase in costs. 

In this study, we retrospectively evaluated the patients referring to hematology outpatient clinic at Izmir Katip Celebi University and Atatürk Training and Research Hospital for preoperative consultation due to elevated levels of PT and aPTT tests.

## MATERIALS AND METHODS

 The study included the patients referring to our hematology clinic for preoperative consultation due to elevated levels of PT and aPTT between January 2016 and January 2017. The exclusion criteria were the presence of a bleeding disorder or a liver disease in the patient, abnormal liver function tests or positive serological tests for hepatitis to rule out any possible liver disease. Similarly, patients with thrombocytopenia (<150 × 10^9^/L) and patients with abnormal thrombocyte aggregation tests were also excluded. The demographic data of the patients, PT, aPTT, and INR values were recorded, retrospectively. 

PT and aPTT levels were studied one more time in the first visit in the hematology outpatient clinic and patients with normal values were excluded. INR, PT, and aPTT values were evaluated according to normal range of the laboratory, and PT ≥ 13.0 seconds, INR ≥1.3 and, aPTT ≥ 36.5 seconds were accepted as high. We also recorded mixing test results and other sophisticated tests to document the reason for elevated coagulation tests. 

Detailed history related to the bleeding risk of the patients, the presence of an abnormal bleeding history in the patient or the family member were searched from the archives of hematology clinic. Patients whose operations were cancelled or postponed were identified, and the delay in the operation was accepted as the time between the planned operation date and the operation date.

**Statistical analysis**

Statistical analysis was performed by SPSS Statistics 20. Continuous data were reported as median (range, minimum and maximum). Qualitative data were given as number of cases and percentages. For statistical analysis, chi square, Fisher's exact, and Mann-Whitney U-test were used. A value of P< 0.05 was considered statistically significant. 

## Results

 Fifty-six patients with high PT/PTT levels were enrolled in the study. 26 (46.4%) patients were male and 30 (53.6%) were female. The median age was 34 (18-75) years. We documented bleeding history in 12 (21.4%) patients. The patients who had a bleeding history revealed mostly abnormal uterine bleeding, epistaxis, and gingival bleeding. Life threatening bleeding was not reported in any of the patients. 

Factor levels were studied in 94.6 % of the patients. The operations were cancelled or postponed at least one month in 38 (67.8%) and 10(17.8%) patients, respectively (Table 1).

**Table 1:** Demographic characteristics of patients

	**All patients** *	**Patients ** **with only ** **high aPTT ** **level **	**Patients ** **with only ** **high PT ** **level**
number	56 (100%)	14 (25%)	40 (71.4%)
Age ( median, range)	34 (18-75)	52 (23-75)	30 (18-72)
Sex ( female/male)	26/30	7/7	21/19
PT (seconds), (median, range)	14.9(10.1- 37.4)	-	15.9 (13.5- 37.4)
INR ( median, range)	1.36 (0.9-3.2)	-	1.4(1.3-3.2)
aPTT (seconds), (median, range)	34 (24-232)	48(38- 232)	
bleeding yes no	12 (21.4%) 44 (78.6%)	3 (21.4%) 11 (78.6%)	9 (22.5%) 31 (77. 5%)
Operations which were cancelled	38 (67.8%)	6 (42.8%)	30 (75%)
Delay in operations ( month (median, range)	3 (1-8)	4 (2-8)	3(1-3)

* Two patients with both elevated PT and aPTT were evaluated in only column called all patients. PT: prothrombin time; aPTT: Activated prothrombin time

We subdivided the cohort as patients with only high PT levels (40 [71.4%]) or patients with only high aPTT levels (14 [25%]). High levels of PT and aPTT coexisted in two patients (3.6%). When we focused on these patients, knee prosthesis replacement and cataract operations of the patients were cancelled, although the family history or personal history did not reveal any abnormal bleeding.

**Patients with only high PT levels**

When we analyzed 40 patients (21 males and 19 females) with only high PT levels, the median age was 30 (range, 18-72) years old. The median PT and INR values were 15.9 seconds (13.5-37.4 seconds) and 1.4 (1.3- 3.2), respectively (Table 1). The number of patients with INR ≥ 1.5 and <1.5 was 14 (35%) and 26 (65 %), respectively (Table 2).

**Table 2:** The demographic characteristics of the patients with elevated PT levels according to INR levesl

	**INR** **≥** **1.5 ( group B)**	**INR<1.5 ( group A)**
Number	14 (35%)	26(65%)
Age	30 ( 19-72)	29 (18-58)
Sex (male/female)	6/8	13/13
Bleeding history	5 (35.7%)	4(15.3%)
Patients in whom factor levels were studied	14 (100%)	23 (88.4%)
Factor VII level (%) (median, range)	23 (5-46)	41 (27-54)

The patients did not have any known bleeding diathesis. In five patients (12.5%), the detailed history revealed mild abnormal bleeding, including epistaxis, menorrhagia, and spontaneous ecchymosis less than 1-2 times a month. No major bleeding that might cause mortality or morbidity in the patients was reported. All the male patients had circumcision, and no abnormal bleeding was documented. Ten (25%) women who gave birth did not describe bleeding during or after childbirth. Teeth extraction was performed in five (12.5%) patients without any bleeding complication. In the family history, one patient revealed undiagnosed probable bleeding diathesis, and the son of one patient was diagnosed as factor VII deficiency. None of the patients had chronic liver disease or vitamin K deficiency. 

The median PT and INR values were 15.3 seconds ( 13.6-17.9) and 1.55 ( 1.4-1.7), respectively in patients with positive personal history for abnormal bleeding, and the median PT and INR values were 16 seconds (13.5-37.4) and 1.4 ( 1.3-3.2) in patients without an abnormal bleeding history. No statistical difference was observed between the two groups. 

The operations were not cancelled in only ten (25%) patients, and the median delay in operations was one month (1-3 months). All the operations, except two, were performed with fresh frozen plasma infusion, and no abnormal bleeding was reported during the perioperative or post-operative period. 

The patients with only high PT levels were subdivided as patients with INR <1.5 (group A, 26 patients [65%]) and patients with INR ≥1.5 (group B, 14 patients [35%]). The demographic characteristics of the patients were documented in Table 2. While personal history revealed an abnormal bleeding history in 15.3% of the patients in group A, 35.7% of the patients in group B reported an abnormal bleeding history, but this difference did not reach statistical significance ( p= 0.14). 

Factor levels were studied in all patients in group B, and the median Factor VII value was 23% (5-46 %). In group A, factor levels were studied in 23 (88.4%) patients, and the median level was 41% (27-54%). There was a statistical difference in terms of factor levels in both groups (p<0.05). The percentage of cancelled operations were statistically similar in group A and group B (76.9% in group A and 71.4% in group B). In both groups, there was no difference in FFP infusion rates, and no abnormal bleeding was reported in operations performed with or without FFP infusion. 

When we subdivided patients with only high PT levels, according to age or sex, we also could not document a statistical difference between groups in terms of INR value, abnormal bleeding, cancelling of the operations, factor levels or FFP fusion rates. 

**Patients with only a PTT levels**

The median age of the 14 patients (7 (%50) females, 7 (%50) males) with only high aPTT levels was 52 (23-75) years old and the median aPTT level was 48% (38-232%). The demographic and laboratory characteristics of the patients are shown in Table 1. 

Three (21.4%) patients had a history of abnormal menstrual bleeding, epistaxis, and gingival bleeding. The patients or their relatives did not have any known bleeding diathesis. Two of the patients with a personal history of abnormal bleeding were diagnosed as factor XI deficiency. Their personal history revealed abnormal gingival and menstrual bleeding. The factor levels were 5% and 2% with aPTT level of 70 and 77.6 seconds, respectively. The teeth extraction procedures were performed with FFP infusion in one of the patients without any complication, but it was cancelled by the dentist in another patient, and medical therapy was preferred. The third patient was also referred to our hematology clinic before teeth extraction. She had abnormal gingival bleeding. The only abnormal factor level, including von Willebrand factor, was factor VIII, and it was 40%. Teeth extraction was performed without any complication, but after the delay of three months. 

All factor levels, including von Willebrand factor, were studied in all patients, except one. This patient did not reveal a personal or family history of abnormal bleeding, and the aPTT level of this patient was 38 seconds. The most common factor deficiency was factor XII deficiency reported in 5 (35.7%) patients. The median levels of factor XII in patients with deficiency were 12 %( 1-27%). The operations were postponed in 42.8 % of the patients, and the median delay in operations was 4 (2-8) months. Abnormal bleeding was not reported in any of the operations which were performed. 

## Discussion

 In Turkey, coagulation tests are widely used without adequate selection among patients to determine the risk of bleeding before the operations. In our study, we analyzed the data of 56 patients who were referred to hematology outpatient clinic due to high PT / aPTT levels. It was observed that 85.6% of electively planned operations were either canceled or postponed for more than a month. However, the personal history of these patients revealed no abnormal bleeding during circumcision in males and labor in females. 

Studies constructed especially before 1990s reported that preoperative coagulation tests might determine the risk of bleeding during or after the operation and these tests were effective in decreasing the rate of abnormal bleeding. But these results could not be confirmed and supported by recent studies and guidelines^3^^, ^^4^^, ^^9^.

Gün et al. analyzed the results of septoplasty, adenoidectomy and tonsillectomy operations in 250 patients and high PT levels were reported in 12.5% of the patients. Intraoperative mild abnormal bleeding was reported in only two patients with high PT levels and no post-operative bleeding was determined ^13^. Moreover, it was also reported in the same study that mild intraoperative bleeding was also documented in 17.4 % of the patients with normal PT levels and post-operative bleeding was observed in 5.5% of these patients with normal coagulation tests^13^. Similarly, we could not document a difference in terms of abnormal bleeding between groups with or without abnormal bleeding tests. Segal et al. investigated the role of coagulation tests performed before diagnostic invasive procedures in determining the risk of abnormal bleeding during or after the procedure. In this review, procedures consisted of angiography, central vein catheterization, biopsy of liver and kidney were evaluated. There was no difference between groups with or without abnormal coagulation tests^14^. It had been suggested that mildly to moderately elevated PT / INR values which were performed before invasive procedures, were insufficient in predicting the risk of bleeding and these values should not be used to decide to give FFP infusion. However, randomized controlled trials are needed to make a final decision^14^. In our study, eight patients were operated with FFP infusion, but when we analyzed the previous procedures of the same patients including circumcision, labor and teeth extraction, no abnormal bleeding were reported without any FFP infusion. Under these circumstances, it is contradictive if FFP infusion was really essential or not. 

A prospective study in this issue consisted of tonsillectomy operations was performed in 1706 children. In this study, abnormal bleeding was related to the technique of the operation, age of the child, but the coagulation tests were thought to be insufficient to predict the risk of bleeding, which was similar to the results of our study^15^. In the literature, the sensitivity and positive predictive value of the coagulation tests was reported as 0.03-0.16 and 0.03-0.14, respectively and similarly, preoperative coagulation tests were not expected as significant to predict the risk of abnormal bleeding ^2^^, ^^15^^-^^17^. 

In the systemic review evaluating the studies consisted of both adult and pediatric patients, it was postulated that elevated coagulation tests were not reliable in determining the operative bleeding risk. But, in this review, it was also reported that the rate of bleeding was as low as 0-0.2 %, and elevated coagulation test results did not result in change in clinical management of the patients^12^. However, in our study although bleeding was not a major complication, high levels of the coagulation test resulted in at least one month delay or cancel of the operation in 85.6 % of the patients. This discrepancy were thought to be explained by the fact that the cases in our study were mostly elective surgeries.

There was conflicting data in the literature related to the role of preoperative coagulation test in predicting the perioperative or post-operative bleeding risk. Most of the studies reported that elevated coagulation tests could not predict the risk of bleeding precisely in different cohorts including different race, age and operations^4^^, ^^15^^, ^^18^^, ^^19^. Beside this data, a study analyzed the tonsillectomy operations in children indicated that high PT and /or a PTT levels might be related to increase in rate of bleeding risk^10^ . In this study, it was reported that, the rate of bleeding was 3.3% in patients with normal PT / a PTT levels while it was 8.7% in patients with high PT / a PTT^10^ although the difference was not statistically significant. 

But, when the studies in the literature were evaluated, it was remarkable that most of the studies were retrospective and methodologically weak because there were different proposals in the guidelines. While, in French and British guidelines, it was not recommended to study coagulation test routinely ^2^^,^^3^, Italian group argued that it might be appropriate to study coagulation test before all operations routinely^10^. When we analyzed the results of our study, it was noteworthy that elevated levels of PT /a PTT were not effective in predicting bleeding risk. Moreover, our study had some limitations. First, it was a retrospective study, so data loss could not be prevented. Second, we did not use a well-conducted questionnaire to search for an abnormal bleeding history in patients and their relatives, but all patients were questioned by an experienced hematologist.

## CONCLUSION

 In conclusion, in the literature, there was lack of randomized prospective studies to predict the role of coagulation tests in predicting risk of bleeding, and most of the studies were consisted of heterogeneous group of patients. Under these circumstances, most of the studies draw the attention to the detailed and well-constructed family and personal history questionnaires. Moreover, coagulations tests should be studied in selected group of patients, especially before operations with low risk of bleeding. Additionally, especially mildly elevated results should be interpreted carefully to decrease the rate of cancellation and delay in operations and unnecessary increase in costs. 
